# High-throughput sheathless and three-dimensional microparticle focusing using a microchannel with arc-shaped groove arrays

**DOI:** 10.1038/srep41153

**Published:** 2017-01-23

**Authors:** Qianbin Zhao, Jun Zhang, Sheng Yan, Dan Yuan, Haiping Du, Gursel Alici, Weihua Li

**Affiliations:** 1 School of Mechanical, Materials and Mechatronic Engineering, University of Wollongong, Wollongong, NSW 2522, Australia; 2 School of Mechanical Engineering, Nanjing University of Science and Technology, Nanjing 210094, China; 3 School of Electric, Computer and Telecommunication Engineering, University of Wollongong, Wollongong, NSW 2522, Australia

## Abstract

Sheathless particle focusing which utilises the secondary flow with a high throughput has great potential for use in microfluidic applications. In this work, an innovative particle focusing method was proposed. This method makes use of a mechanism that takes advantage of secondary flow and inertial migration. The device was a straight channel with arrays of arc-shaped grooves on the top surface. First, the mechanism and expected focusing phenomenon are explained using numerical simulation of the flow field and force balance. A simulation of particle trajectories was conducted as a reference, and then a series of experiments was designed and the effects of changes in particle size, flow rate and quantity of the groove structure were discussed. The microscopic images show that this particle focusing method performed well for different size particles, and the results agreed well with the theory and simulated results. Finally, the channel successfully concentrated Jurkat cells, which showed a good compatibility in the biological assay field. In this work, the arc-shaped groove channel was demonstrated to have the ability to achieve high-throughput, sheathless and three-dimensional particle focusing with simple operations.

Over the past decades, microfluidic technology has attracted a great deal of attention due to its potential applications, especially in the field of biology and diagnostics^1^,^2^,^3^. Microfluidic technologies are suitable for a number of reasons. These include: (1) reduction in the number of samples and reagents necessary; (2) rapid analysis; (3) high sensitivity and resolution in detection; (4) low cost; (5) good portability due to its small size and (6) the potential to be a highly integrated and automated component to meet the experimental requirements^4^,^5^.

According to the focusing principle, the state-of-the-art microfluidic technologies can be categorized as either active or passive focusing methods. Active focusing technologies include acoustophoresis (AP)^6^, dielectrophoresis (DEP)^7^ and magnetophoresis (MP)^8^ which rely on external fields for their functionality. The active focusing technologies can offer precise particle manipulation and high focusing efficiency. A high-throughput acoustophoresis chip (HATC) has also been reported to separate blood cells from the whole blood using a temperature-stabilized standing ultrasonic wave at a flow rate up to 2 L h^−1 ^9. Most of them, however, still have some shortcomings, such as the relatively complicated fabrication of introducing external fields. On the other hand, passive focusing technologies rely entirely on the channel geometry and intrinsic hydrodynamic forces for functionality. Unlike active approaches, passive approaches always offer a remarkably high-throughput with simple set-ups, in which the external fields are not required. These characteristics are extremely useful for some commercial particle and cell focusing applications.

Hydrodynamic focusing using sheath flow is one of the typical passive focusing methods which has been widely adopted in recent years^10^,^11^. The focusing system of sheath flow typically introduces two neighbouring buffer streams with high flow rates to horizontally compress the middle stream containing particles to yield a two-dimensional focusing plane in a narrow width^12^. The typical on-chip single-layer sheath flow focusing system can only concentrate particles in 2D format with a high throughput. Although this problem can be solved using the microfluidic shifting technique to achieve three-dimensional focusing, the logistically burdensome sheath flow still cannot be ignored^13^. Besides the logistical burden, because fluid inertia can cause an uneven pressure gradient within the channel, the focusing profile may be distorted by the high flow rate of the sheath flows and this will obstruct particle focusing^14^,^15^. As a result, a sheathless 3D focusing approach with high-throughputs is more desirable.

Another passive focusing approach, inertial microfluidics, which relies on particle inertial migration phenomenon due to the inertia of flow, is becoming an increasingly popular approach^16^. In a straight channel, inertial migration always works effectively at an intermediate range of flow Reynolds number (~1 < Re < ~100), which is still confirmed as laminar flow and dominated by the viscosity and inertia of the flow^16^,^17^,^18^. Inertial migration caused by this regime can be used to control the internal particles^19^. This approach has attracted a great deal of attention due to its excellent compatible characteristics in terms of both yielding and precision. Inertial microfluidics in a straight channel, however, has some limitations: (1) the cross-section of the channel (aspect of ratio) must be matched in size for effective focusing functionality; (2) particles must pass through a long enough distance in order to move laterally to the equilibrium positions; (3) there are always several equilibrium positions in one channel at the same time^20^,^21^,^22^. In order to solve these problems, secondary flow is introduced by transforming the geometry of the straight channels by adding a curved portion or disturbance obstacles^23^,^24^,^25^. The secondary flow induced by these structures exerts a secondary flow drag force on the particles, which: (1) eliminates the limitations of channel size, (2) enhances the efficiency of particle focusing and (3) reduces the requirement of long distance for sufficient displacement to equilibrium positions^16^. The flow rates of various particle focusing methods are listed in Table 1.

In this paper, a sheathless, high-throughput and three dimensional particle focusing device consisting of a low aspect ratio straight channel (AR = 0.2) and an arc-shaped groove array pattern on the surface is presented. In this channel, the secondary drag force drives particles to a new equilibrium position with good focusing performance at a moderate flow rate. Simulation by finite element software is carried out and calculated numerically to describe the focusing mechanism. This focusing approach has been demonstrated by a series of experiments on different size particles (10 μm, 13 μm and 24 μm) as well as Jurkat cells. All particles and cells of all sizes focus well at the moderated flow rates (Reynolds number), and this agrees with the mechanism and simulations. The effect of groove structure quantity on particle focusing is also discussed and investigated by observing the performance of particle focusing after travelling through different numbers of groove structures. This innovative focusing approach shows good focusing performance which is useful for many biological and diagnostic devices.

## Theory

### Inertial migration

When particles are dispersed randomly at the entrance of a straight channel, the inertia of the fluid and particles causes the particles to migrate laterally to several equilibrium positions within the cross-section of the channel after travelling a long enough distance^26^,^27^. This interesting phenomenon has been widely recognised by the counteraction of two dominated inertial effects: (1) the shear gradient lift force *F*_Ls_, originating from the curvature of the fluid velocity profile and its interaction with the particle, which directs particles away from the centre of the channel, and (2) the wall lift force *F*_Lw_, as a result of the flow field interaction between suspended particles and adjacent walls, which repels particles away from the wall^28^.

**Figure 1:**
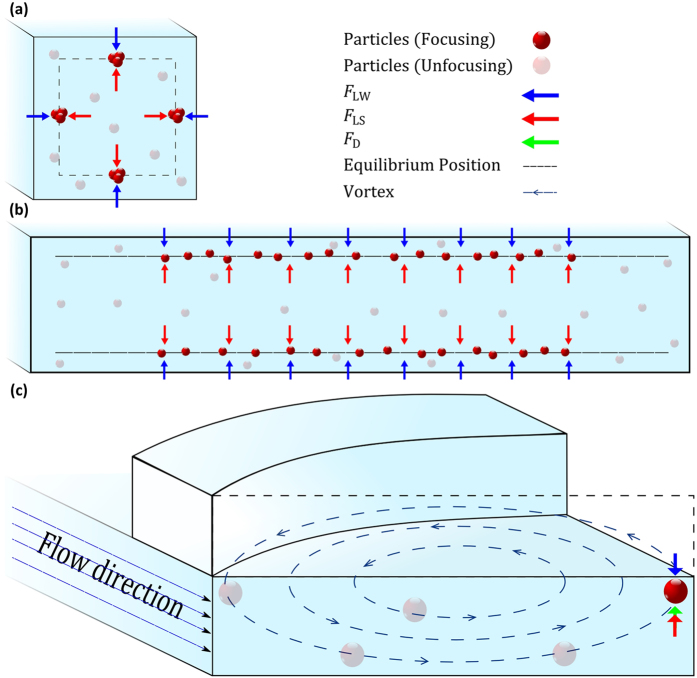
Schematic drawing of the equilibrium positions in a straight channel with different geometric cross-sections. (**a**) In a square channel, the particles focus at four equilibrium positions facing the centre of four walls. (**b**) In a straight channel with a low aspect ratio rectangular cross-section (AR = 0.2), particles form two long bands along the top and bottom of the channel. (**c**) In a straight channel (AR = 0.2) combined with arc-shaped groove arrays, transverse secondary flow is induced by this asymmetric structure within the cross-sections, and thus a huge vortex rotates upwards and downwards. As a result, the particles are directed to a position where the inertial lift force counteracts the secondary flow drag force to generate a balance.

In a channel, the inertial lift force exerts onto flowing particles and forms some equilibrium positions, which is closely related to the geometry of the channel’s cross-section. Due to limitations in microfabrication, the cross-sections of most channels utilised in microfluidic devices are rectangular. When particles travelling in a square straight channel (AR = height/width = 1), they form four equilibrium positions centred at the faces of the channel at a finite Reynolds number (Fig. 1a)^29^. As the aspect ratio (AR) decreases, the number of equilibrium positions reduces to two which are located along the longer faces as the velocity profile varies from a sharp parabolic curve to a blunted one^15^. As the AR decreases continuously to 0.2, however, the two equilibrium positions gradually spread out to two relatively wide bands parallel with longer faces of the channel (Fig. 1b). The particles migrate to the top and bottom of the channel along shorter faces because the parabolic velocity profile between two longer faces is sharp, while it is difficult to see the particles focusing along the longer face with an extremely weak shear gradient lift force due to the blunt velocity profile between two shorter faces^30^.

The net inertial lift force *F*_*L*_ acting on a small rigid sphere is expressed by Asmolov as^31^:


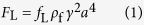


This expression can be simplified as:


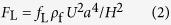



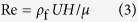



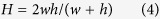


where *ρ*_**f**_, *U, γ* and *μ* are fluid density, maximum velocity, shear gradient and dynamic viscosity, respectively. *a* is the particle diameter, and *H* is the hydraulic diameter of the channel (*w* is the width and *h* is the height of the channel). The lift coefficient *f*_L_ is a function of the lateral position of particles *x* and the Reynolds number Re^31^,^32^.

### Secondary flow

By adding arc-shaped groove arrays on top of the straight channel (AR = 0.2), a secondary flow appears when the fluid flow tends to be trapped in the groove structure due to a pressure gradient in the transverse direction. The secondary flow vorticity induced by the circulation of fluid can influence the particle distribution within cross-sections (Fig. 1c). It is expected that the flow will be required to turn to a larger extent, creating a larger transverse pressure gradient driving the secondary flow and generate a secondary flow drag force that is strong enough to manipulate the particles^15^. To generate a strong enough secondary flow, the ratio of the height of the arc-shaped groove arrays and main channel is set as ~1.

**Figure 2:**
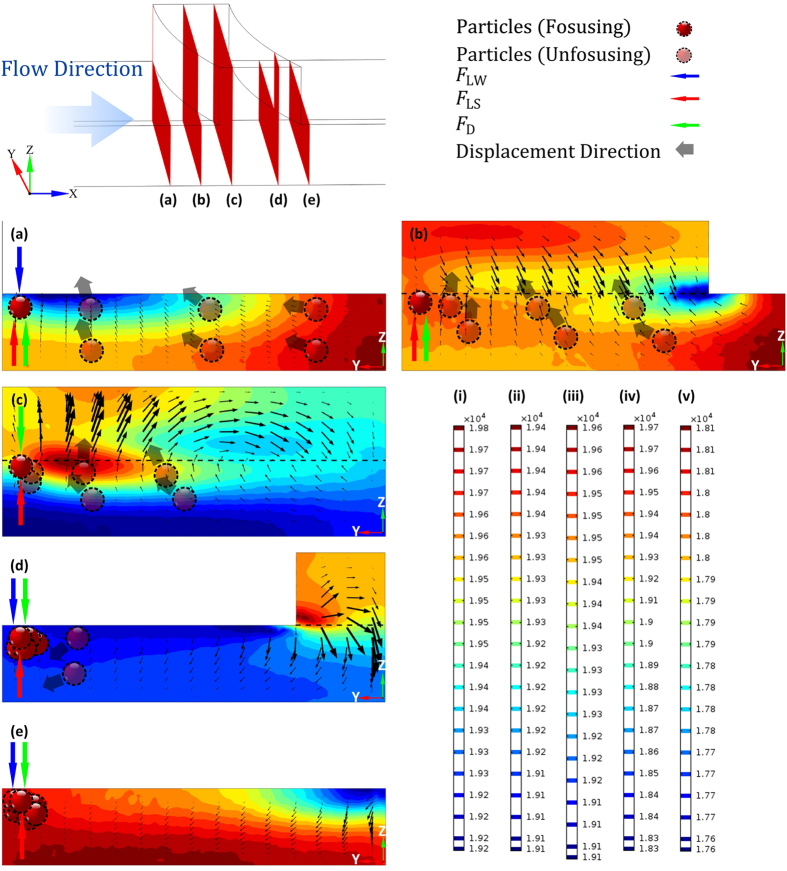
Finite element analysis of flow field and characterization of rotational vorticity at modulated flow rate within cross-sections obtained by COMSOL Multi-physics 5.1. A 3D model of a straight channel (AR = 0.2) with a single arc-shaped groove has been imported into COMSOL to investigate the alteration of flow field. The dotted line is used to distinguish the interface between the main channel and the arc-shaped groove. (**a–e**) These five schematic diagrams (a combination of the velocity arrow plot and the pressure contour plot) show the streamline displacement and pressure alternation. The vector arrows represent the flow velocity and the arrow size is proportional to the magnitude of the flow velocity. The expected process of particle focusing is depicted in these five images. (i–v) are the corresponding colour bars of (**a–e**) to show the colour scale. The unit on the colour bars is Pa.

Because the arc-shaped groove arrays are composed of the same arc-grooves, the computational fluid dynamics analysis can be simplified by simulating only one section with a single characteristic groove structure (Fig. 2). The vorticity in the flow is created within the cross-sections, as the flowing fluid is deflected by the arc-shaped groove (Fig. 2a–e). Thus, the secondary flow gradually transports the fluid to shift laterally along the vorticity rotational direction within the cross-sections. In this process, the direction of rotation and the magnitude of the secondary flow are the important factors which determine particle displacement. In these five cross-section plots, it can be seen that the direction of the vorticity in the main channel (except the groove part) is constantly directed in a (Y) direction, and the fluid in the arc-shaped groove part will simultaneously re-circulate in the opposite (−Y) direction due to the conservation of mass. The whole helical vorticity pattern is shown in Fig. 1c.

At a low Reynolds number (typically in microfluidic systems), the drag force originating from the transverse fluid flow which is exerted on the small rigid spherical particle can be expressed as follows:


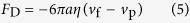


where *a* is the radius of the particle, *η* is the viscosity of the fluid, *v*_f_ is the dynamics velocity of the fluid, and *v*_p_ is the velocity of the particle^33^.

The process of focusing is shown in Fig. 2a–e. After being distributed randomly at an entrance, the particles locate along the top and bottom surfaces of the channel and form two wide focusing bands due to the inertial migration. When particles are flowing through the parts with the groove structure, the transverse vorticity imposes vertical and lateral drag forces which cause a displacement (shown as grey arrows in Fig. 2) of the particles within the cross-sections. Because the vorticity in the main channel (apart from the groove part) is half of the vorticity circulation (another half occurs in the groove part), the corresponding drag force (*F*_D_) on particles is constantly directed in the Y direction and the particles are gradually gathered to one side of the channel. Simultaneously, the shear gradient lift force (*F*_Ls_, shown as bright red arrows in Fig. 2) and the wall lift force (*F*_Lw_, shown as bright blue arrows in Fig. 2) push particles away from the centre of the channel and repel the particles away from the wall of the channel, which will help them to achieve a balance and locate at an equilibrium position. It can be seen that the secondary flow vorticity is not symmetric and the region near the left wall (where particles gathered) is more stable than the other regions (as shown in the simulation results Fig. 2a–e), because it can be seen that the variation of the arrow size which depicts the magnitude of the flow field is smaller than other parts. This also means that particles tend to locate at the left region. This also shows that a trap tendency appears at the left upper corner where the inertial lift force (consisting of the shear gradient lift force *F*_Ls_ and the wall lift force *F*_Lw_) counteracts the drag force (*F*_D_) and achieves a balance. In addition, if the particle is large enough, particles will not be trapped in the groove part. As a result, this mechanism means that a three-dimensional, high-throughput particle focusing method can be realized using this arc-shaped groove channel at a moderated flow rate.

## Results and Discussion

### Simulation of particle trajectories

**Figure 3:**
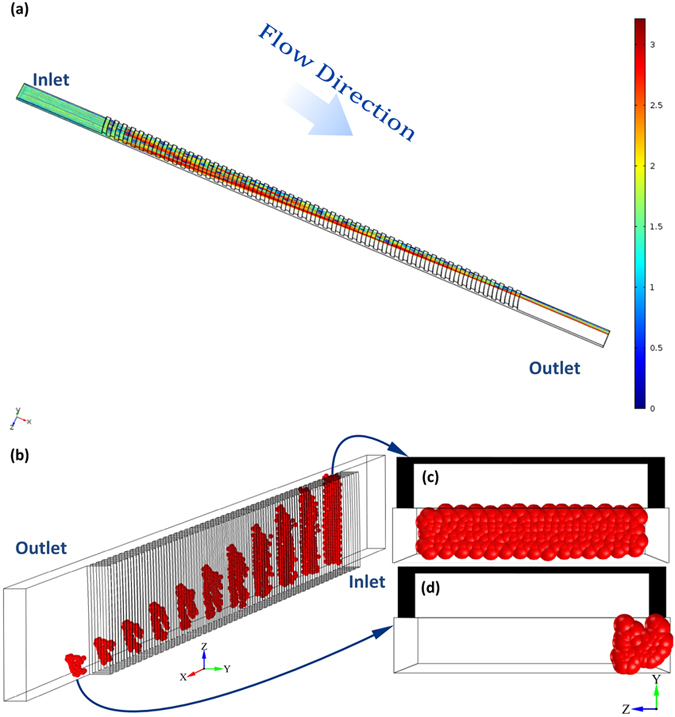
Simulation obtained by COMSOL Multi-physics 5.1. (**a**) The image shows the whole arc-shaped groove channel, where particles (not to scale) concentrated to one side of the channel. The unit on the scale bar is m/s. (**b**) We calculated the distribution diagram of particles after travelling past 0, 5, 10, …, 50 groove structures and indicated by Poincare maps. (**c**) Particle distribution within the first cross-section. (**d**) Particle distribution within the last cross-section.

Figure 3a shows the simulation of particle trajectories in the arc-shaped groove channel, where 200 13 μm particles were evenly distributed form the inlet at a flow velocity of 2.29 m/s (Re = 160.4). The performance of particle focusing was demonstrated by the contractive particle trajectories, where the colour of the trajectories denotes the particle velocity.

Figure 3b shows the Poincare maps of particle distributions in different cross-sections in the channel. The portion with the groove structure was divided into 10 sections with 11 cross-section plots. Within the first cross-section plot, particles were evenly distributed, but those particles within the following cross-section plots tended to concentrate step by step to one side. Finally, after travelling through the whole portion, all these particles gathered together at a small region within the last cross-sections.

### Effects of the channel Reynolds number

**Figure 4:**
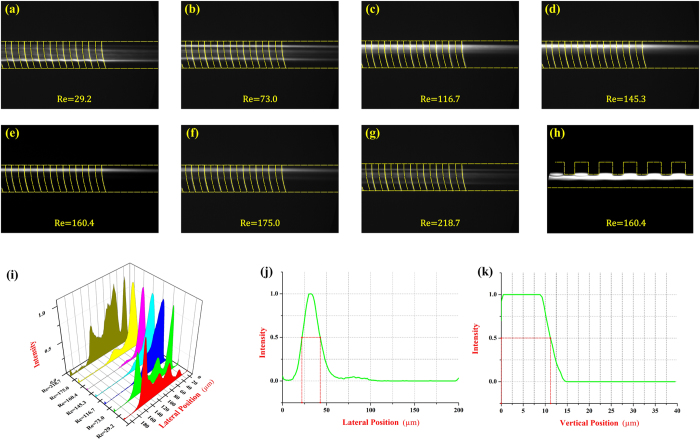
(**a**–**g**) The optical microscopy images captured by the CCD camera illustrate the focusing performance of 13 μm particles in the arc-shaped groove channel. The Reynolds numbers range from 29.2 to 218.7 and the corresponding flow rates are 200 μl/min, 500 μl/min, 800 μl/min, 1000 μl/min, 1100 μl/min, 1200 μl/min and 1500 μl/min. (**h**) The side view of the optical image of particle focusing pattern shows the distribution of 13 μm particles in vertical direction. (**i**) The particle focusing positions at different Reynolds numbers are measured and depicted by the normalized intensity profiles. (**j**) Determination of focusing band width by normalized intensity profile. (**k**) The normalized intensity profile of particle focusing positions in vertical direction.

To evaluate the effect of flow rate (channel Reynolds number) on the particle focusing performance, a series of validation experiments at various flow rates was conducted. Here, the 13 μm particle suspensions were injected into the arc-shaped groove channel form the inlet. When the flow rate increased from 200 μl/min to 1500 μl/min (Reynolds number from 29.2 to 218.7), different particle focusing patterns and qualities occurred near the outlet of the arc-shaped groove channel shown by Fig. 4a–g. At relatively low flow rates (200 μl/min and 500 μl/min), the focusing patterns were still inconspicuous and shaped as wide bands (Fig. 4a,b) but the focusing performance was improved when the flow rate increased. The fluorescent images (Fig. 4c–e) show that the particle trajectories tend to concentrate at one side of the channel until the flow rate increased to 1100 μl/min (Re = 160.4). The reason is that the magnitude of secondary flow drag force was the crucial factor to the particle focusing performance and a higher flow rate was able to induce a stronger secondary flow within the main channel. As a result, the induced stronger secondary flow exerted a stronger lateral drag force on the particles, which enabled them to assemble into a small region quickly and efficiently. When the flow rate exceeded 1200 μl/min (Re = 175.0), however, the stabilized concentrated particle focusing pattern was destroyed due to the violent interaction between particles in the narrow region (Fig. 4f,g). Since the particles in this series of experiments were 13 μm in diameter and the height of channel was 40 μm, they tended to interact with each other when gathering together. Figure 4i is a wall diagram including the particle focusing patterns at different flow rates (200 μl/min to 1500 μl/min) and was established by measuring the particle fluorescent intensity. This diagram shows that the 13 μm particles focusing efficiency was proportional to the flow rate and reached its optimal performance at 1100 μl/min (Re = 160.4). To assess the particle focusing performance numerically, the focusing band width was determined through measuring the length between two points where the normalized intensity profile crossed the 50% threshold. And focusing was attained when the band width was <2 times the diameter of the focused particles^34^. Figure 4j, the intensity profile at 1100 μl/min (Re = 160.4), shows that the length between two points was measured <25 μm, which means that particle focusing was achieved. On the other hand, to investigate the focusing type of this method, the PDMS channel could be located vertically onto the microscope stage in order to provide a clear enough side view of the arc-shaped groove channel (Fig. 4h). In the plot, particles were found to concentrate along the top of the main channel (the interface between the groove arrays and the main channel) and had a trend to be trapped into the groove structure. Because the size of particles (13 μm) was large enough compared with the gap of the groove (50 μm), particles could be retained in the fluid stream line and not be trapped by the groove structure. The side view of normalized intensity profile (Fig. 4k) showed that the particle distribution in vertical direction located in one streamline approximately as well. As a result, this particle focusing approach in the arc-shaped groove channel could be considered as a three-dimensional focusing.

### Effects of the quantity of groove structure

**Figure 5:**
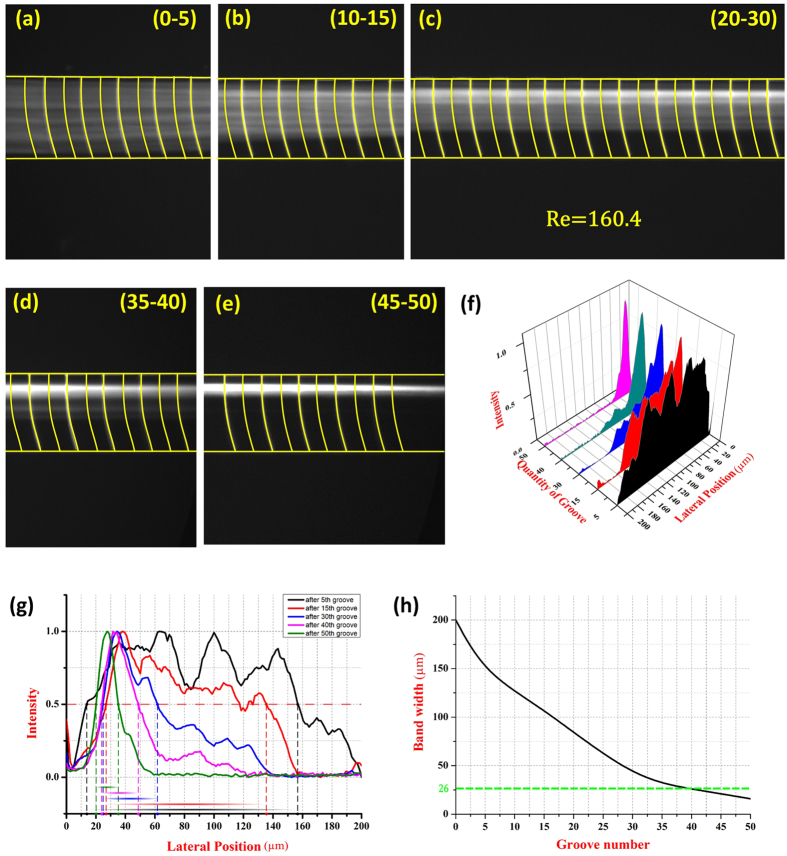
(**a**–**e**) The optical microscopy images of the focusing process of 13 μm particles at different segments of the arc-shaped groove channel (total of 50 grooves). (**f**) Particle focusing positions at different segments of the channel shown by a normalized intensity profile. (**g**) The measured particle focusing band width from normalized intensity profile. (**h**) Particle focusing band width under different numbers of groove structures.

For this particle focusing method, particles were concentrated by the secondary flow which was induced by the groove structure. So it was necessary for particles to pass through plenty of groove structures to achieve adequate lateral displacements for an effective particle focusing. In order to investigate the influence of quantity of the groove structure on particles focusing, 13 μm particle suspensions were injected form the inlet at a flow rate of 1100 μl/min (Re = 160.4) and fluorescent images of the particle flowing pattern after travelling different quantity of the groove structure were captured (Fig. 5a–e). The microscopic images show that the focusing performance was improved significantly as particles passed more quantity of the groove structure. The particles were gradually pushed to one side and located in a line observing through the (0–5), (10–15), (20–30) and (35–40) segment of the channel. Finally, all the particles were able to be concentrated at the equilibrium position which agreed well with the simulation results achieved (Fig. 5e). The normalized intensity profile (Fig. 5f) showed a comparison of particle focusing patterns at different segments in the channel. According to the particle focusing assessment above, an effective particle focusing could be attained in the channel with more than approximate 40 groove structures when the focusing band width was <2 times the diameter of the focused particles (13 μm) (Fig. 5g,h).

### Effects of the particle size

**Figure 6:**
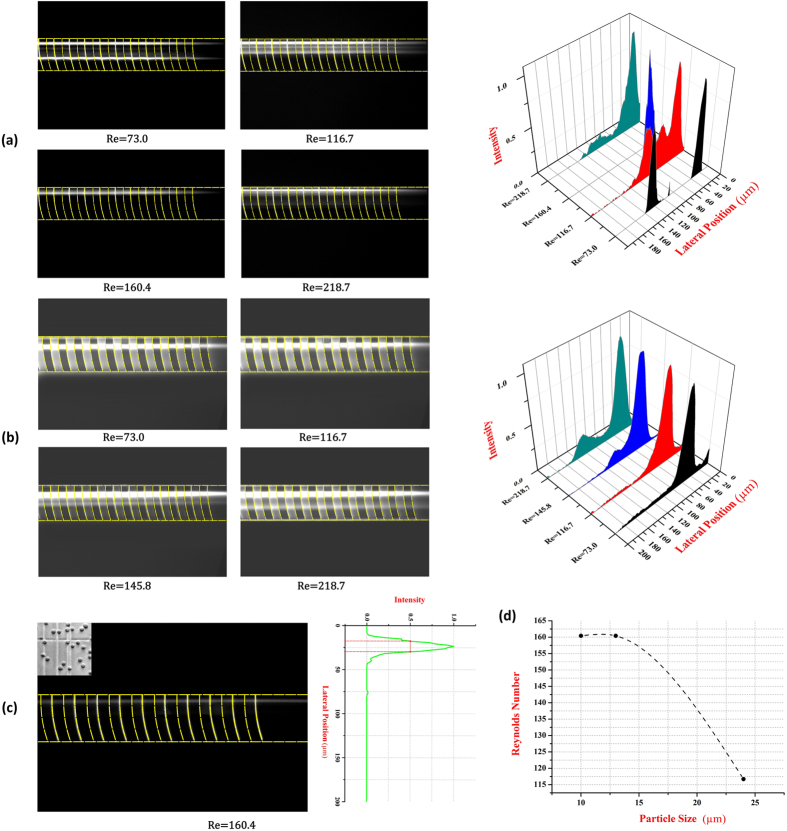
Focusing patterns of different particles and Jurkat cells. (**a**) The optical microscopy images of 10 μm particles focusing at different flow rates respectively and the corresponding normalized intensity profile. (**b**) Images of 24 μm particles focusing at different flow rates respectively and the corresponding normalized intensity profile. (**c**) Focusing pattern of Jurkat cells at a flow rate of 1100 μl/min (Re = 160.4) and the corresponding normalized intensity profile. And the optical microscopy of Jurkat cells is shown at the corner. (**d**) The predicted relationship between particle size and corresponding optimal focusing Reynolds number.

Figure 6a shows that 10 μm particles focused in the same way as 13 μm particles, and they were located precisely at the equilibrium position at the flow rate of 1100 μl/min (Re = 160.4). However, when the 24 μm particles travelled in the channel, the focusing pattern differed from the 10 μm and 13 μm particles (Fig. 6b). The 24 μm particles could concentrate at one side of the channel at only 500 μl/min (R_e_ = 73.0) and 800 μl/min (Re = 116.4), while the focusing pattern was loose at 1000 μl/min (Re = 145.8). This phenomenon could be explained that the secondary flow drag force exerted on particles was proportional to particles size according to equation (5). Particles with diameter of 24 μm could receive a lager drag force than 13 μm particles, and tended to focus at lower flow rates. However, the 24 μm particles could not focus effectively at a flow rate of 1000 μl/min, because there was a greater possibility that the larger size could induce a stronger interaction between the particles and reduce the focusing performance. The intensity profiles of fluorescent particles show the focusing performance of 10 μm and 13 μm respectively.

In conclusion, the critical Reynolds number (Re) of optimal focusing performance in the channel was predicted to be inversely proportional to the size of the particle (Fig. 6d). And there was also a critical particle size range which is appropriate for particle focusing in the selected channel. In the arc-shaped groove channel, the critical particle size range was predicted from 10 μm to 24 μm. It was not easy to identify the accuracy range, because the commercial microparticles had a relatively large coefficient of variation (CV), such as a CV 16% of 13 μm particles, and this error would influence the experiments.

To confirm the feasibility of this focusing method on cells, 14 μm Jurkat cell suspensions were introduced into the arc-shaped groove channel. According to the secondary flow drag force equation (5), the forces (*F*_D_ and *F*_L_) exerted on the cells were expected to be similar to the forces on 13 μm particles. The microscopy image (Fig. 6c) shows that the arc-shaped groove channel performed well on focusing Jurkat cells at 1100 μl/min (Re = 160.4) and the normalized intensity profile also demonstrates that most Jurkat cells concentrated at one side of the channel. The experiment result agreed well with the focusing mechanism and the experimental results of rigid polystyrene microparticles.

## Materials and Methods

### Design and fabrication of the microchannel

**Figure 7:**
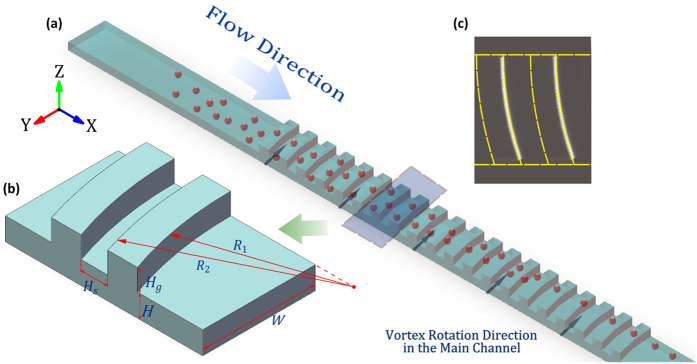
(**a**) The schematic drawing of the microfluidic device and particle focusing pattern (not to scale). (**b**) The 3D model of the arc-shaped groove structure shows the size parameters (zoom-in). (**c**) The image of the microchannel with the arc-shaped grooves captured by a CCD camera.

Figure 7a is a schematic drawing of the microchannel with the arc-shaped grooves utilised in experiments. The microchannel is a stacked structure which consists of a main straight channel (AR = 0.2) and arc-shaped groove arrays. The main channel width (*W*) is 200 μm with a height (*H*) of 40 μm and a total length (*L*) of 7 mm. The groove arrays are located on the top of the main channel between 1 and 6 mm downstream from the inlet; they are composed of 50 arc-shaped grooves with 50 μm spacing (*H*_s_) between them. The groove is arc-shaped with a small curvature of 600 μm (*R*_1_) and a large curvature of 650 μm (*R*_2_). The height of the groove arrays (*H*_g_) is 38 μm (Fig. 7b).

A double-layer silicon master was fabricated by the two-step photolithography technique on a silicon wafer. The first layer was processed as the main channel and the second layer was processed as the arc-shaped groove arrays aligned to locate on top of the first layer (the main channel). The first layer photoresist (PR) (SU-8 2050, Microchem Corp., Newton, MA) was spun on a clean silicon wafer through a three-step coating cycle (500 rpm for 20 s, 2000 rpm for 20 s, and 4000 rpm for 40 s). Then, the silicon wafer with coated photoresist was heated at the temperature of 65 °C for 2 minutes and 95 °C for 7 minutes. After the first heating, the silicon wafer was exposed by UV light through the first photomask and subsequently heated by the second two-step hard baking (at 65 °C for 3 minutes and 95 °C for 7 minutes). Afterwards, the silicon wafer was developed in the SU-8 developer solution and rinsed with isopropyl alcohol (IPA). The remaining photoresist pattern attaching onto the wafer formed the first layer (the main channel). In the second-step photolithography, the second spin-coating photoresist on the previous mould was processed according to the first-layer process. Then, the second photomask was carefully aligned to the processed photoresist pattern (the first layer), and UV light was used to expose onto the second layer photoresist through the second photomask. After heating and developing the pattern, the double-layer mould was processed with trichlorosilane to deposit a monolayer onto the surface.

A mixture of poly(dimethylsiloxane) (PDMS) and curing agent (Dow Corning, Midland, MI) in a ratio of 10:1 was poured onto the double-layer mould, degassed and then cured in a convection oven at 65 °C for 2 hours. The cured PDMS was then peeled off from the mould and punched through the inlet and outlet holes with a custom needle tip. Finally, The PDMS replica was sealed with a glass slide after treatment in oxygen plasma (PDc-002, Harrick Plasma, Ossining, NY) for 3 minutes.

### Preparation of particles and cells

Green fluorescent polystyrene particles with a diameter of 9.9 μm (CAT. NO. G1000, 5% CV), red fluorescent polystyrene particles with a diameter of 24 μm (CAT. NO. 36-5, 12% CV) and 13 μm (CAT. NO. 36-4, 16% CV) were purchased from Thermo Fisher Scientific, and then suspended in deionized (DI) water. The concentrations of the particle in these suspensions were ~5 × 10^4^ counts/ml, ~2 × 10^5^ counts/ml and ~5 × 10^4^ counts/ml respectively. To prevent the aggregation, sedimentation, and adhesion to the microchannel walls, 0.1% w/v Tween 20 (Sigma-Aldrich, Product NO. P9416) was added to the particle suspensions^24^,^35^,^36^.

Jurkat cell lines were cultured in RPMI medium 1640 (Gibco) supplemented with 100 units/ml aqueous penicillin, 100 μg/ml streptomycin and 10% fetal bovine serum at 37 °C in an atmospheres of 100% humidity and 5% CO_2_. The cell diameter ranges from 10 and 15 μm. For better observation and being captured using a CCD camera, the Jurkat cells were stained with Calcein AM (Thermo Fisher Scientific) for 30 minutes. The concentration of Jurkat cells used in the experiment was ~5 × 10^4^ counts/ml in the suspensions.

### Experimental setup

To observe and record the particle focusing phenomenon, the microfluidic device was placed on an inverted microscope (CKX41, Olympus, Japan). A CCD camera (Optimos, Q-imaging, Australia) was used to observe and capture the fluorescent particle trajectory images, which were then post-processed and analysed using the image processing software, Q-Capture Pro 7 (Q-imaging, Australia). The exposure time was set as 10 ms. Particle fluorescent trajectories were obtained by stacking 50 consecutive frames in order to measure the focusing position of the fluorescent particles.

### Numerical simulation

A numerical simulation was conducted to calculate the flow field characteristics and particle trajectories to investigate and predict the focusing phenomenon with finite element software (COMSOL Multi-physics 5.1, Burlington, MA). A 3D model of the microchannel with 50 arc-shaped grooves was built using the modelling function of COMSOL. A laminar flow model was then added to the component to calculate the flow field. In laminar flow physics, the boundary condition was set as no slip and the physical property of fluid was set as a steady incompressible flow. At the inlet, the normal inflow velocity was set as 2.29 m/s (the corresponding flow rate and Reynolds number were 1100 μl/min and Re = 160.4 respectively) and the pressure at the outlet was set as 0 Pa. Afterwards, a model of particle tracing for fluid flow was added to the same component and coupled with the previous physics to predict the particle trajectories in the microchannel. At the entrance, 200 13 μm particles with a density of 1050 kg/m^3^ were released randomly along the channel width. The time range was set from 0 to 0.019 s with an interval time of 10^−5^ s, in time settings. For the numerical solution, the order of finite element was set as P1 + P1, the default setting of COMSOL Multiphysics and the mesh was set as the free tetrahedral at finer level (1488007 elements). The equations to govern steady incompressible flow are as below:

N-S equation:


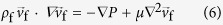


Continuity equation:


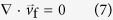


Non-slip boundary condition:


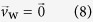


where 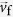 is the velocity vector and 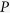 is the pressure of flow field, respectively; 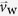 is the fluid velocity vector at the channel walls; ∇ is the Nabla operator: 
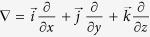
, and 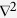 is the Laplace operator: 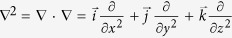.

For this particle focusing method, the secondary flow drag force works dominantly and guides the particle distribution within the channel, while the inertial affects only act as an assisting role to balance particles when they reach the equilibrium position. Meanwhile, the inertial lift force is much weaker compared with the secondary flow drag force, especially along longer faces of channel for the blunt velocity profile. As a result, we neglect the intricate inertial lift force and only consider the secondary flow drag force to reduce the computational time. On the other hand, to simplify the simulation, the fluid-structure interactions were neglected^37^,^38^,^39^. In this work, the drag force dominates the external force exerted on particles at low Reynolds numbers, so the governing equations are according to equation (5).

## Conclusions

A passive particle focusing approach has been developed in this paper by utilising an arc-shaped groove channel. The benefits of this approach are that different size particles can be focused in a three-dimensional type, all in a high-throughput manner, without the requirement of sheath flow. The focusing mechanism was explained and demonstrated by numerical simulation of the flow field. As the inertial focusing performance in a low aspect ratio channel (AR = 0.2) was not effective, the arc-shaped groove arrays were located on the top of the straight channel to induce a secondary flow rotating within the channel cross-sections and a new equilibrium position of particles was formed under the balance between the secondary flow drag force and inertial lift force. Meanwhile, through verification experiments, the focusing approach showed a good applicability for different size particles and cells (Jurkat cells). In conclusion, this research provides a sheathless and high-throughput particle focusing method in three-dimensions, which has the potential to be used for particle or cell concentration, filtration and flow cytometry.

## Additional Information

**How to cite this article**: Zhao, Q. *et al*. High-throughput sheathless and three-dimensional microparticle focusing using a microchannel with arc-shaped groove arrays. *Sci. Rep.* **7**, 41153; doi: 10.1038/srep41153 (2017).

**Publisher's note:** Springer Nature remains neutral with regard to jurisdictional claims in published maps and institutional affiliations.

## Figures and Tables

**Table 1:** List of the flow rates of different particle focusing methods.

Classification	Method	Focusing mechanism	Flow rate
Active	Acoustophoresis^40^	Standing surface acoustic waves (SSAW)	10 μl/min
	Dielectrophoresis^41^	Externally controlled positive Dielectrophoresis	0.01 μl/min
Passive	Hydrodynamics^13^	Dean flow and sheath flow	330 μl/min
	Inertial microfluidics in a straight channel^42^	Inertial migration	140 μl/min
	Inertial microfluidics in a curved channel^17^	Inertial migration and Dean flow	~20 μl/min
	Inertial microfluidics in a step channel^15^	Secondary flow and Inertial migration	~295 μl/min
	This	Secondary flow and Inertial migration	~1100 μl/min

## References

[b1] Adams A. A. . Highly efficient circulating tumor cell isolation from whole blood and label-free enumeration using polymer-based microfluidics with an integrated conductivity sensor. Journal of the American Chemical Society 130, 8633–8641 (2008). 18557614 10.1021/ja8015022 PMC2526315

[b2] Nagrath S. . Isolation of rare circulating tumour cells in cancer patients by microchip technology. Nature 450, 1235–1239 (2007). 18097410 10.1038/nature06385 PMC3090667

[b3] Hoshino K. . Microchip-based immunomagnetic detection of circulating tumor cells. Lab on a Chip 11, 3449–3457 (2011). 21863182 10.1039/c1lc20270g PMC3379551

[b4] Whitesides G. M. The origins and the future of microfluidics. Nature 442, 368–373 (2006). 16871203 10.1038/nature05058

[b5] Bhagat A. A. S. . Microfluidics for cell separation. Medical & biological engineering & computing 48, 999–1014 (2010). 20414811 10.1007/s11517-010-0611-4

[b6] Wang Z. & Zhe J. Recent advances in particle and droplet manipulation for lab-on-a-chip devices based on surface acoustic waves. Lab on a Chip 11, 1280–1285 (2011). 21301739 10.1039/c0lc00527d

[b7] Çetin B. & Li D. Dielectrophoresis in microfluidics technology. Electrophoresis 32, 2410–2427 (2011). 21922491 10.1002/elps.201100167

[b8] Forbes T. P. & Forry S. P. Microfluidic magnetophoretic separations of immunomagnetically labeled rare mammalian cells. Lab on a Chip 12, 1471–1479 (2012). 22395226 10.1039/c2lc40113d

[b9] Adams J. D. . High-throughput, temperature-controlled microchannel acoustophoresis device made with rapid prototyping. Journal of Micromechanics and Microengineering 22, 075017 (2012).

[b10] Simonnet C. & Groisman A. Two-dimensional hydrodynamic focusing in a simple microfluidic device. Applied Physics Letters 87, 114104 (2005).

[b11] Anna S. L., Bontoux N. & Stone H. A. Formation of dispersions using “flow focusing” in microchannels. Applied physics letters 82, 364–366 (2003).

[b12] Knight J. B., Vishwanath A., Brody J. P. & Austin R. H. Hydrodynamic focusing on a silicon chip: mixing nanoliters in microseconds. Physical Review Letters 80, 3863 (1998).

[b13] Nawaz A. A. . Sub-micrometer-precision, three-dimensional (3D) hydrodynamic focusing via “microfluidic drifting”. Lab on a Chip 14, 415–423 (2014). 24287742 10.1039/c3lc50810b PMC3989543

[b14] Park H. Y. . Achieving uniform mixing in a microfluidic device: hydrodynamic focusing prior to mixing. Analytical chemistry 78, 4465–4473 (2006). 16808455 10.1021/ac060572n

[b15] Chung A. J., Gossett D. R. & Di Carlo D. Three dimensional, sheathless, and high‐throughput microparticle inertial focusing through geometry‐induced secondary flows. Small 9, 685–690 (2013). 23143944 10.1002/smll.201202413

[b16] Di Carlo D. Inertial microfluidics. Lab on a Chip 9, 3038–3046 (2009). 19823716 10.1039/b912547g

[b17] Di Carlo D., Irimia D., Tompkins R. G. & Toner M. Continuous inertial focusing, ordering, and separation of particles in microchannels. Proceedings of the National Academy of Sciences 104, 18892–18897 (2007). 10.1073/pnas.0704958104 PMC2141878 18025477

[b18] Sudarsan A. P. & Ugaz V. M. Multivortex micromixing. Proceedings of the National Academy of Sciences 103, 7228–7233 (2006). 10.1073/pnas.0507976103 PMC1464325 16645036

[b19] Amini H., Lee W. & Di Carlo D. Inertial microfluidic physics. Lab on a Chip 14, 2739–2761 (2014). 24914632 10.1039/c4lc00128a

[b20] Zhou J. & Papautsky I. Fundamentals of inertial focusing in microchannels. Lab on a Chip 13, 1121–1132 (2013). 23353899 10.1039/c2lc41248a

[b21] Lee M. G., Choi S. & Park J.-K. Inertial separation in a contraction–expansion array microchannel. Journal of Chromatography A 1218, 4138–4143 (2011). 21176909 10.1016/j.chroma.2010.11.081

[b22] Zhang J. . Fundamentals and applications of inertial microfluidics: a review. Lab on a Chip 16, 10–34 (2016). 26584257 10.1039/c5lc01159k

[b23] Lee M. G., Choi S. & Park J.-K. Rapid laminating mixer using a contraction-expansion array microchannel. Applied Physics Letters 95, 051902 (2009).

[b24] Zhang J., Li M., Li W. & Alici G. Inertial focusing in a straight channel with asymmetrical expansion–contraction cavity arrays using two secondary flows. Journal of Micromechanics and Microengineering 23, 085023 (2013).

[b25] Amini H. . Engineering fluid flow using sequenced microstructures. Nature communications 4, 1826 (2013). 10.1038/ncomms2841 23652014

[b26] Segre G. Radial particle displacements in Poiseuille flow of suspensions. Nature 189, 209–210 (1961).

[b27] Segre G. & Silberberg A. Behaviour of macroscopic rigid spheres in Poiseuille flow Part 2. Experimental results and interpretation. Journal of Fluid Mechanics 14, 136–157 (1962).

[b28] Rafeie M., Zhang J., Asadnia M., Li W. & Warkiani M. E. Multiplexing slanted spiral microchannels for ultra-fast blood plasma separation. Lab on a Chip 16, 2791–2802 (2016). 27377196 10.1039/c6lc00713a

[b29] Choi Y.-S., Seo K.-W. & Lee S.-J. Lateral and cross-lateral focusing of spherical particles in a square microchannel. Lab on a Chip 11, 460–465 (2011). 21072415 10.1039/c0lc00212g

[b30] Zhang J., Li W., Li M., Alici G. & Nguyen N.-T. Particle inertial focusing and its mechanism in a serpentine microchannel. Microfluidics and Nanofluidics 17, 305–316 (2014).

[b31] Asmolov E. S. The inertial lift on a spherical particle in a plane Poiseuille flow at large channel Reynolds number. Journal of Fluid Mechanics 381, 63–87 (1999).

[b32] Bhagat A. A. S., Kuntaegowdanahalli S. S. & Papautsky I. Inertial microfluidics for continuous particle filtration and extraction. Microfluidics and nanofluidics 7, 217–226 (2009). 10.1039/b908271a 19789752

[b33] Gerlach T. Microdiffusers as dynamic passive valves for micropump applications. Sensors and Actuators A: Physical 69, 181–191 (1998).

[b34] Martel J. M. & Toner M. Inertial focusing dynamics in spiral microchannels. Physics of Fluids (1994-present) 24, 032001 (2012). 10.1063/1.3681228 PMC3311666 22454556

[b35] Li M. . A simple and cost-effective method for fabrication of integrated electronic-microfluidic devices using a laser-patterned PDMS layer. Microfluidics and nanofluidics 12, 751–760 (2012).

[b36] Yan S. . Isolating plasma from blood using a dielectrophoresis-active hydrophoretic device. Lab on a Chip 14, 2993–3003 (2014). 24939716 10.1039/c4lc00343h

[b37] Nama N. . Numerical study of acoustophoretic motion of particles in a PDMS microchannel driven by surface acoustic waves. Lab on a Chip 15, 2700–2709 (2015). 26001199 10.1039/c5lc00231a PMC4465433

[b38] Nama N., Huang P.-H., Huang T. J. & Costanzo F. Investigation of acoustic streaming patterns around oscillating sharp edges. Lab on a Chip 14, 2824–2836 (2014). 24903475 10.1039/c4lc00191e PMC4096312

[b39] Muller P. B., Barnkob R., Jensen M. J. H. & Bruus H. A numerical study of microparticle acoustophoresis driven by acoustic radiation forces and streaming-induced drag forces. Lab on a Chip 12, 4617–4627 (2012). 23010952 10.1039/c2lc40612h

[b40] Shi J., Mao X., Ahmed D., Colletti A. & Huang T. J. Focusing microparticles in a microfluidic channel with standing surface acoustic waves (SSAW). Lab on a Chip 8, 221–223 (2008). 18231658 10.1039/b716321e

[b41] Chu H., Doh I. & Cho Y.-H. A three-dimensional (3D) particle focusing channel using the positive dielectrophoresis (pDEP) guided by a dielectric structure between two planar electrodes. Lab on a Chip 9, 686–691 (2009). 19224018 10.1039/b812213j

[b42] Park J.-S., Song S.-H. & Jung H.-I. Continuous focusing of microparticles using inertial lift force and vorticity via multi-orifice microfluidic channels. Lab on a Chip 9, 939–948 (2009). 19294305 10.1039/b813952k

